# Empfehlungen zur Auswahl von Zielparametern und Prozessempfehlungen bei audiologisch-technischen Funktionsprüfungen des Cochlea-Implantats: Erarbeitet von der Arbeitsgruppe ERA (AG-ERA) der ADANO in Kooperation mit dem Fachausschuss „Cochlea-Implantate und implantierbare Hörsysteme“ der DGA. Bestätigt vom Vorstand der ADANO am 31.01.2025.

**DOI:** 10.1007/s00106-025-01628-x

**Published:** 2025-05-23

**Authors:** Alexander Müller, M. Blümer, O. C. Dziemba, A. Elsholz, L. Fröhlich, U. Hoppe, D. Polterauer, T. Rahne, T. Steffens, M. Walger, T. Weißgerber, T. Wesarg, S. Zirn, T. Rader

**Affiliations:** 1 Hörzentrum Berlin, Klinik für Hals‑, Nasen-, und Ohrenheilkunde, Kopf- und Halschirurgie, Plastische Operationen, Zentrum für Hörimplantate, Vivantes Klinikum im Friedrichshain, Landsberger Allee 49, 10249 Berlin, Deutschland; 2 Klinik- und Poliklinik für Hals‑, Nasen- und Ohrenheilkunde, Universitätsklinikum Hamburg-Eppendorf, Hamburg, Deutschland; 3 Klinik und Poliklinik für Hals‑, Nasen‑, Ohrenkrankheiten, Kopf- und Halschirurgie, Universitätsmedizin Greifswald, Greifswald, Deutschland; 4 Klinik und Poliklinik für Hals-Nasen-Ohren-Heilkunde, Universitätsklinikum Bonn, Bonn, Deutschland; 5 Hals‑, Nasen‑, OhrenKlinik, Kopf- und Halschirurgie, Universitätsklinikum Erlangen, Erlangen, Deutschland; 6 Klinik und Poliklinik für Hals-Nasen-Ohrenheilkunde, Klinikum der Ludwig-Maximilians-Universität München, München, Deutschland; 7 Hallesches Hör- und ImplantCentrum, Universitätsklinik und Poliklinik für Hals- Nasen-Ohrenheilkunde, Kopf- und Halschirurgie, Universitätsmedizin Halle (Saale), Halle (Saale), Deutschland; 8 Klinik und Poliklinik für Hals-Nasen-Ohren-Heilkunde, Universitätsklinikum Regensburg, Regensburg, Deutschland; 9 Klinik und Poliklinik für Hals‑, Nasen‑, Ohren-Heilkunde, Kopf- und Hals-Chirurgie, Audiologisches Zentrum, Universitätsklinikum, Köln, Deutschland; 10 Klinik für Hals‑, Nasen‑, Ohrenheilkunde, Universitätsmedizin, Goethe-Universität Frankfurt am Main, Frankfurt am Main, Deutschland; 11 Universitätsklinikum Freiburg, Medizinische Fakultät, Albert-Ludwigs-Universität Freiburg, Freiburg, Deutschland; 12 Peter-Osypka-Institut für Medizintechnik, Hochschule für Technik, Wirtschaft und Medien, Offenburg, Deutschland

**Keywords:** Qualitätssicherung, Minimalstandard, Messverfahren, Evozierte Potentiale, Impedanzen, Quality assurance, Minimum standard, Measurement methods, Evoked potentials, Impedances

## Abstract

Die kontinuierliche Kontrolle der technischen und physiologischen Funktion von Cochlea-Implantaten (CI) stellt einen zentralen Baustein im gesamten Versorgungsprozess dar. Trotz weltweiter Bestrebungen zur Vereinheitlichung der Verfahren zeigen sich nach wie vor erhebliche Unterschiede zwischen den CI-versorgenden Einrichtungen – insbesondere hinsichtlich der eingesetzten Methoden, ihrer praktischen Umsetzung und der Festlegung aussagekräftiger Zielgrößen. Für eine verlässliche Qualitätssicherung und verbesserte Vergleichbarkeit ist ein einheitlicher, strukturierter Prüfprozess erforderlich. Vor diesem Hintergrund wurde in einem offenen Konsensverfahren der Arbeitsgruppe Elektrische Reaktionsaudiometrie (AG-ERA) der ADANO, gemeinsam mit dem Fachausschuss „Cochlea-Implantate und implantierbare Hörsysteme“ der DGA, ein Minimalstandard für die audiologisch-technische Funktionsprüfung von CI entwickelt. Dieser definiert grundlegende Anforderungen an Durchführung und Dokumentation und dient als praxisnahe Empfehlung für CI-versorgende Einrichtungen. Ziel ist eine standardisierte, nachvollziehbare Vorgehensweise, die die interdisziplinäre Zusammenarbeit verbessert, die Versorgungsqualität erhöht und eine strukturierte, langfristig optimierte Betreuung von CI-Tragenden ermöglicht.

## Abstract (en)

Continuous monitoring of the technical and physiological function of cochlear implants (CI) is a central part of the care process. Despite worldwide efforts to standardise procedures, there is still considerable variation between CI centres, particularly in terms of the methods used, their practical implementation and the definition of meaningful target parameters. A standardised structured test procedure is needed for reliable quality assurance and better comparability. Against this background, the ADANO Working Group for Evoked Response Audiometry (AG-ERA), in close cooperation with the Cochlear Implants and Implantable Hearing Systems Committee of the German Society of Audiology (DGA), developed a minimum standard for audiological and technical functional testing of CIs in an open consensus process. This standard defines basic requirements for performance and documentation and serves as a practical recommendation for CI centres. It is intended to improve interdisciplinary cooperation, increase the quality of care and enable structured long-term optimised care for CI patients.

## Präambel

Während des gesamten CochIea-Implantat(CI)-Versorgungsprozesses muss eine möglichst detaillierte Information über die technische und physiologische Funktion des Implantats zur Verfügung stehen. Zusätzlich soll die korrekte Lage des Elektrodenträgers regelmäßig überprüft werden. Zu diesem Zweck werden i. d. R. intra- und postoperativ umfangreiche technische und elektrophysiologische Messungen mit dem CI durchgeführt [11, 42, 57, 58]. Sie sind fester Bestandteil der audiologischen Diagnostik.

Trotz internationaler Bestrebungen zur Vereinheitlichung der Prozesse besteht immer noch eine große Heterogenität in den Abläufen der CI-versorgenden Einrichtungen bei der Auswahl und Nutzung geeigneter Methoden sowie der damit verbundenen Zielparameterwahl [2, 3]. Deshalb halten die Autoren einen standardisierten Prozess der audiologisch-technischen Funktionsprüfungen des CIs, die Festlegung durchzuführender Messverfahren und die Definition relevanter Zielparameter für notwendig. Als Ergebnis eines offenen Verfahrens innerhalb der Arbeitsgruppe Elektrische Reaktionsaudiometrie (AG-ERA) der ADANO (Arbeitsgemeinschaft Deutschsprachiger Audiologen, Neurootologen und Otologen) der DGHNO-KHC (Deutsche Gesellschaft für HNO-Heilkunde, Kopf- und Halschirurgie) und in enger Kooperation mit dem Fachausschuss „Cochlea-Implantate und implantierbare Hörsysteme“ in der DGA (Deutsche Gesellschaft für Audiologie) wurde ein Minimalstandard zur Durchführung audiologisch-technischer Funktionsprüfungen von CIs während des gesamten Versorgungsprozesses festgelegt und konsentiert.

Dieser Minimalstandard gilt nicht nur für die CI-versorgenden Einrichtungen, sondern bezieht explizit auch die CI-Firmen mit ein. Es ist essenziell, dass herstellerseitig geeignete Mess- und Auswerteverfahren zur Verfügung gestellt und in die jeweiligen Anwendersoftware-Lösungen integriert werden. Durch diese Maßnahmen soll gewährleistet werden, dass die Messwerte der definierten Zielparameter zuverlässig erfasst, reproduzierbar ausgewertet und über verschiedene Versorgungseinrichtungen hinweg vergleichbar gemacht werden können.

Der Einsatz von Impedanzmessungen der Elektroden [1, 29, 30, 37, 40, 41, 55, 57], Messungen der Impedanz- oder Spannungsmatrix [5, 6, 17, 22, 25, 45, 54, 59], elektrisch evozierten Stapediusreflexen [8, 16, 31, 53, 56, 57], Summenaktions- und Hirnstammpotentialen [7, 9, 13, 14, 18–21, 26, 27, 31, 32, 34–36, 38, 39, 43, 47, 51, 52, 60], Potentialen des auditorischen Kortex [15, 27, 33, 46, 48, 58] und der extra- und intracochleären Elektrocochleographie während und nach der Elektrodeninsertion [10, 23, 24, 28] bietet eine große Bandbreite möglicher zu messender Zielgrößen. Zudem gibt es verschiedene herstellerspezifische Optionen und Implementierungen der o. g. Messverfahren in der jeweiligen Anwendersoftware. Einige dieser Verfahren sind derzeit jedoch nicht bei allen Herstellern standardmäßig integriert. Daher enthalten diese Empfehlungen auch Angaben für den jeweiligen Messprozess. Die definierten Zielparameter- und Prozessempfehlungen sollen einerseits die profunde Beurteilung der Messergebnisse ermöglichen, um damit auch die regelhafte technische und physiologische Funktion des Implantats zu bestätigen. Andererseits sollen sie zur Sicherung der Versorgungsqualität [49] gemäß der aktuell gültigen S2k-Leitlinie zur CI-Versorgung der Arbeitsgemeinschaft der Wissenschaftlichen Medizinischen Fachgesellschaften (AWMF-Register-Nr.: 017–071) [4] und des Weißbuchs „Cochlea-Implantat (CI)-Versorgung“ der DGHNO-KHC [12] beitragen und sollen ggf. auch für das DGHNO-CI-Register Verwendung finden [50].

Die Anwendung weiterer Methoden und die Auswahl zusätzlicher Zielparameter zur Untersuchung individueller Fragestellungen, z. B. bei relativer Kontraindikation zur CI-Versorgung (s. [4], S. 33), sollen durch diese Empfehlung explizit nicht eingeschränkt werden.

## Empfehlungen

**Abb. 1:**
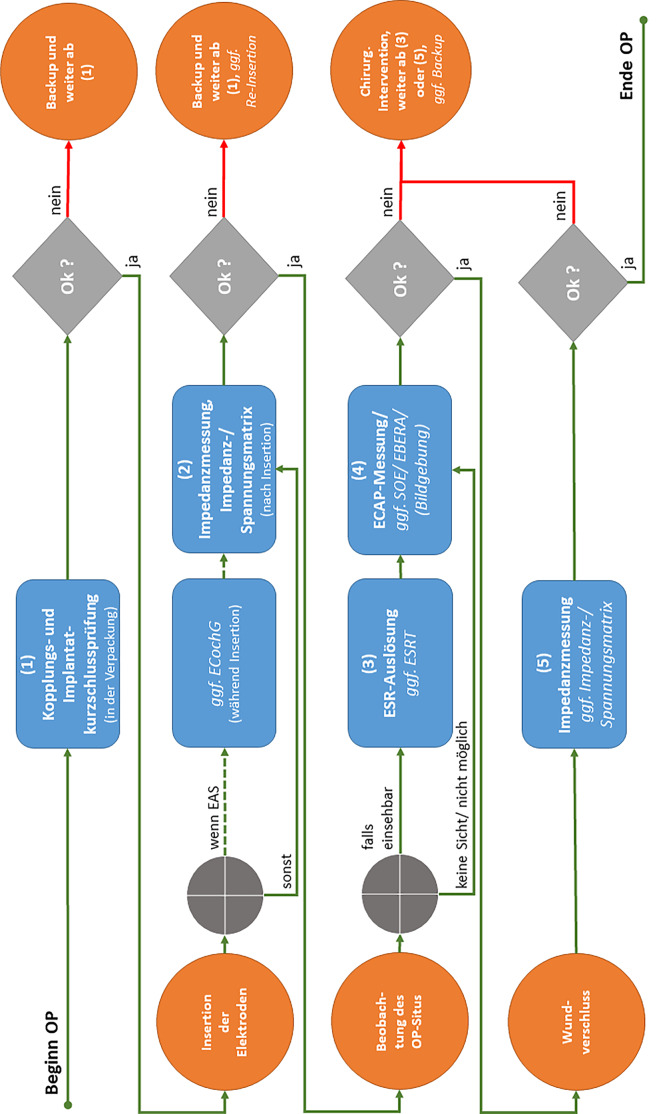
Prozessbeschreibung bei audiologisch-technischen Funktionsprüfungen des Cochlea-Implantats während der Operation: Nach der Kopplungs- und Implantatkurzschlussprüfung in der Verpackung und/oder in Natriumchlorid(NaCl)-Lösung *(1)* erfolgt die Insertion des Elektrodenträgers, bei resthörerhaltenden Operationen (EAS) ggf. mit Echtzeit-Elektrocochleographie(ECochG)-Monitoring. Anschließend werden die elektrischen (technischen) Funktionskontrollen des Cochlea-Implantats mittels Impedanzmessung und Messung der Impedanz- oder Spannungsmatrix durchgeführt (SCINSEV) *(2)*. Bei festgestelltem Elektrodenproblem soll unmittelbar der möglichen Ursache nachgegangen werden und im Bedarfsfall ein Backup-Implantat zum Einsatz kommen. Wenn möglich, erfolgt im Anschluss unter Beobachtung des Operationssitus die elektrische Auslösung der Stapediusreflexe (ESR), ggf. mit Bestimmung der Auslöseschwelle (ESRT) *(3)* und die Messung der elektrisch evozierten Summenaktionspotentiale (ECAP) *(4)*. Bei schwierigen Fragestellungen oder unklaren Ergebnissen werden zusätzlich elektrisch evozierte Hirnstammpotentiale (EBERA) und/oder die Spread-of-Excitation (SOE) gemessen, ggf. wird eine intraoperative Bildgebung durchgeführt. Falls erforderlich, folgt eine chirurgische Intervention und/oder die Verwendung des Backup-Implantats. Nach Wundverschluss wird die Impedanzmessung wiederholt *(5)*

Wenn im Sinne dieser Empfehlungen audiologisch-technische Funktionsprüfungen des CIs oder elektrophysiologische Funktionsprüfungen des Hörsystems während und nach der Elektrodeninsertion durchgeführt werden, sollen für verschiedene Fragestellungen die in Tab. 1 aufgeführten Methoden (Spalte 1) genutzt und jeweils die genannten Zielparameter (Spalte 3) sowie während der Operation entsprechend der Prozessbeschreibung aus Abb. 1 gemessen, bestimmt und berichtet werden. Ebenfalls sollen die aufgeführten Prozessempfehlungen (Spalte 4) eingehalten werden.

**Tab. 1:** Zielparameter- und Prozessempfehlungen der unterschiedlichen Messmethoden

Methode	Fragestellung(en)	Zielparameterempfehlung	Prozessempfehlung	Regelhafter Befund (Minimalkonsens)
STANDARD-VERSORGUNG (Vorhaltung notwendig)				
***Kopplungs- und Implantatkurzschlussprüfung***	– Prüfung auf bidirektionale telemetrische Datenübertragung – Kontrolle der spezifikationsgerechten Funktion des Implantats inkl. der Elektrodenkontakte – Hinweise auf Defekte des Implantats	Spulen-Implantat-Kopplung [ja/nein] Impedanzen [*Z* in kΩ]	*Messung in allen verfügbaren Stimulationsmodi an allen Elektrodenkontakten des Implantats* *präOP: *vor Beginn der Operation in der sterilen Verpackung (alternativ: Überprüfung in NaCl-Lösung direkt vor Insertion) *intra-/postOP:* Monitoring der Kopplung während der gesamten audiologisch-technischen Diagnostik	**(1) stabile Kopplung und bidirektionale Datenübertragung** **(2) keine Kurzschlüsse zwischen den Elektrodenkontakten** z. B. in [3, 57, 58]
***Impedanzmessung der Elektroden***	– Kontrolle der spezifikationsgerechten Funktion des Implantats – Überprüfung aller Elektrodenkontakte auf dem Elektrodenträger sowie der externen Referenzelektrode(n) – Hinweise auf Defekte des Implantats	Impedanzen [*Z* in kΩ] Massepfadimpedanz [*Z* in kΩ]	*Messung in allen verfügbaren Stimulationsmodi an allen Elektrodenkontakten des Implantats* *intraOP:* direkt nach Insertion (ggf. nach Elektrodenfixierung) *und *nach Wundverschluss *postOP:* regelmäßig (z. B. vor jeder/m Anpassung/ Fitting)	**(1) kein Hinweis auf offene Stromkreise (keine auffälligen Impedanzen)** **(2) keine Kurzschlüsse zwischen den Elektrodenkontakten** **(3) Impedanzen aller intracochleären Elektroden im Erwartungsbereich des jeweiligen Implantatmodells oder Herstellers** z. B. in [1, 29, 30, 37, 40, 41, 55, 57]
***Messung der stimulationsstrominduzierten nichtstimulierenden Elektrodenspannung (SCINSEV)***	– Kontrolle der spezifikationsgerechten Funktion des Implantats – Schätzung der Elektrodenposition(en) und Identifizierung einer Fehllage wie „tip fold-over“, „base kinking“ – Derzeit im Fokus der Forschung sind Einführtiefe und Lage des Elektrodenträgers, z. B. bei Migration des Elektrodenträgers [5, 6]	Spannung pro Stromeinheit [*R*_n,m_ in kΩ]	*Messung der SCINSEV und Darstellung in einer 12* *×* *12-, 16* *×* *16- oder 22* *×* *22-Matrix (je nach Implantatmodell)* *intraOP:* nach Elektrodenträgerinsertion (ggf. nach Elektrodenträgerfixierung) *postOP:* regelmäßig im Verlauf	**(1) kein Hinweis auf offene Stromkreise (keine auffälligen Impedanzen)** **(2) keine Kurzschlüsse zwischen den Elektrodenkontakten** **(3) kontinuierlicher Abfall der Spannung pro Stromeinheit an der Aufzeichnungselektrode mit zunehmender Entfernung von der Stimulationselektrode** z. B. in [17, 22, 25, 45, 54, 59]
***Elektrisch evozierte Stapediusreflexe (ESR)***	– Physiologische Funktionsprüfung der Signalleitung (untere Hörbahn – Olivenkomplex – Hirnstammebene) und Reflexauslösung durch den *N. facialis* – Bestätigung der vollständigen Insertion bzw. Ausschluss einer möglichen Fehlinsertion des Elektrodenträgers – Bestimmung der oberen Grenze des elektrischen Dynamikbereiches (Über- oder Unterstimulation) – Plausibilitätskontrolle von Stimulationsparametern und ECAP	Auslösen des Reflexes [auslösbar/nicht auslösbar] ESR-Auslöseschwellen [*Q* in nC]	*Stimulation auf mehreren über den Elektrodenträger verteilten Elektrodenkontakten (basal – medial – apikal)* *intraOP:* visuelle Detektion (ipsilateral) nach erster Impedanzmessung und Messung der SCINSEV unter Sicht (intraoperativer Situs) bei elektrischer Stimulation (z. B. durch Eingabeln der Auslöseschwelle) *postOP:* bei Bedarf und wenn durchführbar Messung über ein Impedanzaudiometer bei direkter elektrischer Reizung (ipsilateral) an ausgewählten Elektroden oder Bestimmung der Auslöseschwellen im Freifeld	**(1) Reflex auslösbar und als Bewegung der/des Stapessehne und/oder -köpfchens identifizierbar** z. B. in [8, 16, 31, 53, 56, 57]
***Elektrisch evozierte Summenaktionspotentiale des Hörnervs (ECAP)***	– Reizantwortschwellendiagnostik/Schwellenprofil (Ausgangsbefunde und Daten für die CI-Anpassung) – Verlaufskontrolle der neuronalen Parameter/retrocochleäre Diagnostik bei unklarer Integrität des Hörnervs – Lageprüfung des Elektrodenträgers (z. B. Ausschluss von „tip fold-over“, Elektrodenmigration)	Nachweisbarkeit der ECAP [ja/nein] ECAP-Schwellen [*Q* in nC] ECAP-Wachstumsfunktionen (AGF), N1–P1-Amplitude [*U* in µV] Absolutlatenzen von N1 und P1 [*t* in µs] SOE-Profil, N1–P1 Amplituden [*U* in µV]	*Bestimmung der ECAP-AGF, ECAP-Latenzen sowie ECAP-Schwellen auf allen intracochleären Elektroden (12–22, je nach Implantatmodell)* *Messung von ein oder mehreren SOE-Profilen an mehreren Elektroden (apikal), je nach Implantatmodell und Elektrodenträger* *intraOP:* nach erfolgter Impedanzmessung, ggf. auch nach Elektroden-Konditionierung, bei Bedarf im Anschluss SOE-Messung *postOP:* regelmäßig (vor/bei Anpassung/Fitting) nach Impedanzmessung, bei Bedarf SOE-Messung	**(1) ECAP an allen Elektroden nachweisbar** **(2) ECAP-Schwellen liegen im Erwartungsbereich des jeweiligen Implantatmodells (abhängig von den Werten der Stimulationsparameter)** z. B. in [7, 19, 26, 31, 32, 36, 38, 47] **(3) bei SOE unter Beibehaltung einer definierten Prüfelektrodenposition (Prüf- und Ableitelektrode) nimmt der Einfluss der Verschiebung der Maskierungsposition (Maskerelektrode) entlang des Elektrodenträgers kontinuierlich ab (abhängig von den Werten der Stimulationsparameter und vom jeweiligen Implantatmodell)** z. B. in [9, 14, 21, 35, 39, 43]
***Frühe elektrisch evozierte Potentiale (FEEP, Technik: EBERA)***	– Retrocochleäre Diagnostik bei auditorischer Synaptopathie/Neuropathie (AS/AN) sowie unklarer Integrität des Hörnervs und unteren Hirnstamms, Detektion von Reifungs- und Degenerationsprozessen – Beurteilung der Reizverarbeitung und -weiterleitung in besonderen Fällen, d. h. wenn keine ECAP-Messung möglich (bei erworbenen Veränderungen des Innenohrs, z. B nach chronischen Entzündungen, traumatischen Veränderungen) – Reizantwortschwellendiagnostik	Absolutlatenzen der Wellen eIII und eV sowie Interpeaklatenz (IPL) in Abhängigkeit von der Reizstärke: [*t* in ms] Kleinster Reiz mit identifizierbarer und reproduzierbarer Reizantwortschwelle [*Q* in nC] Potentialamplituden [*U* in nV]	*Potentialmessung auf ausgewählten (intracochleären) Elektrodenkontakten oder Bereichen, Detektion der Potentiallatenzen und Bestimmung der Reizantwortschwelle* *intraOP:* bei Bedarf Messung über ein AEP/ERA-System nach Impedanz‑/ESR-/ECAP-Messung, Position der Ableitelektroden: Cz oder Fpz vs. Mastoid oder Ohrläppchen (kontralateral) bzw. im unteren Bereich des *M. sternocleidomastoideus *(ipsilateral) *postOP: *bei Bedarf Messung über ein AEP/ERA-System, Position der Ableitelektroden: Cz oder Fpz vs. Mastoid oder Ohrläppchen (ipsi- und kontralateral)	**(1) Hirnstammpotentiale überschwellig auslösbar, Wellen eIII und eV gut identifizierbar, Absolutlatenzen sowie Interpeaklatenzen liegen im Erwartungsbereich (abhängig von den Werten der Stimulationsparameter)** **(2) FEEP-Schwellen liegen im Erwartungsbereich (abhängig von den Werten der Stimulationsparameter)** z. B. in [13, 18, 20, 27, 34, 51, 52, 58, 60]
BEI EAS/HYBRID-VERSORGUNG (Vorhaltung im Einzelfall nützlich)				
***Elektrocochleographie (ECochG)***	– Kontrolle und Überwachung (Monitoring) der cochleären Funktion von Haarsinneszellen sowie afferenten Hörnervenfasern (Restgehörerhalt) vor, während sowie nach der Insertion des Elektrodenträgers – Kontrolle der Haarzellfunktion während und nach der Implantation	Nachweisbarkeit der 3 Signalkomponenten der ECochG (CM, CAP, SP), mind. „cochlear microphonic“ (CM) Bestimmung der (größten) CM-Amplitude [*U* in µV]	*Akustische Stimulation, z.* *B. bei 500* *Hz („tone burst“) mit ca. 40* *dB SL sowie Potentialmessung auf ausgewählten Elektrodenkontakten* *intraOP:* bei Bedarf Ableitung der ECochG *extracochleär:* Elektrode am runden Fenster (vor Insertion) oder auf dem Promontorium (während der Insertion), *intracochleär:* apikale Elektrode; jeweils in Referenz zu einer Ableitelektrode (Cz oder Fpz) *postOP:* bei Bedarf Ableitung der ECochG *intracochleär:* apikale Elektrode (22 oder 1 je nach Implantatmodell); jeweils in Referenz zu einer Ableitelektrode (Cz oder Fpz)	**(1) kein (rascher) Amplitudenabfall in der ECochG während der Insertion des Elektrodenträgers** **(2) Intra- und postOP stabile CM-Amplituden** z. B. in [10, 23, 24, 28]
OPTIONAL (vorwiegend wissenschaftlich interessant)				
***Späte elektrisch evozierte Potentiale des auditorischen Kortex (SEEP, Technik: ECERA)***	– Integritätsprüfung der Hörbahn bis zur Ebene der Hirnrinde – Beurteilung der Hörbahnreifung auf kortikaler Ebene	P1–N1–P2–N2-Komplex [*t* in ms]	*Potentialmessung auf ausgewählten Elektrodenkontakten bzw. Bereichen, Detektion der Potentiallatenzen und Bestimmung der Reizantwortschwelle* *postOP:* bei Bedarf Messung über ein AEP/ERA-System, Position der Ableitelektroden: Cz oder Fpz vs. Mastoid oder Ohrläppchen (ipsi- und kontralateral)	**(1) Kortikale Potentiale überschwellig auslösbar, P1–N1–P2–N2-Komplex gut identifizierbar, Absolutlatenzen sowie Interpeaklatenzen liegen im Erwartungsbereich (abhängig von den Werten der Stimulationsparameter)** z. B. in [15, 27, 33, 46, 48, 58]

*AEP* Akustisch Evozierte Potentiale, *AGF* Amplitude Growth Function*, CAP* Compound Action Potential, *CI* Cochlea-Implantat*,* *CM* Cochlear Microphonic*,* *Cz* Elektrodenposition am Vertex*, EAS* Elektrisch-akustische Stimulation, *EBERA* Electrically-evoked Brainstem Response Audiometry, *ECAP* Electrically-evoked Compound Action Potential, *ECERA* Electrically-evoked Cortical Response Audiometry, *ECochG* Elektrocochleographie, *ERA* Elektrische Reaktionsaudiometrie, *ESR* Electrically-evoked Stapedius Reflex, *ESRT* Electrically-evoked Stapedius Reflex Threshold, *FEEP* Frühe Elektrisch Evozierte Potentiale, *Fpz* frontozentrale Elektrodenposition*, NaCl* Natriumchlorid, *SCINSEV* Stimulation-Current-Induced Non-Stimulating Electrode Voltage, *SEEP* Späte Elektrisch Evozierte Potentiale, *SOE* Spread-of-Excitation, *SP* Summationspotential

Die Sicherung einer möglichst hohen Qualität der Untersuchungsergebnisse wird durch die Sicherstellung der fehlerfreien Funktion aller medizintechnischen Gerätekomponenten, die Schaffung optimaler Messbedingungen (angemessener Untersuchungsraum) durch Artefaktreduktion bei möglichst geringen Störschallpegeln und/oder Beseitigung/Abschirmung von elektromagnetischen Störfeldern, die Minimierung des Restrauschens und der Reststörung (z. B. durch Entspannung/Schlaf/Sedierung/Narkose der zu untersuchenden Person) und die fachliche Qualifikation und Erfahrung der Anwendenden gewährleistet.

## Begriffsbestimmungen

### Stimulationsstrominduzierte nichtstimulierende Elektrodenspannung.

Indirektes Maß für die elektrische Feldausbreitung entlang des Elektrodenträgers und aus der *Cochlea* heraus (im Text auch mit SCINSEV [44] abgekürzt), gemessen als Spannung pro Stromeinheit und dargestellt in einer *m* × *n*‑Matrix, d. h. die durch den Eingangsstrom *I* an einer Stimulationselektrode (*m*) induzierte elektrische Spannung *U* als Potentialdifferenz zwischen einer (nichtstimulierenden) Aufzeichnungselektrode (*n*) und einer Masseelekrode, angegeben in der Maßeinheit kΩ mit

$$\boldsymbol{R}_{m,n}=\frac{\boldsymbol{U}_{n}}{\boldsymbol{I}_{m}}$$

### Reizantwortschwelle.

Kleinste angelegte elektrische Ladung *Q* mit (noch) identifizierbarer und reproduzierbarer Reizantwort (in der Tab. 1 auch als Auslöseschwelle oder Schwelle bezeichnet) als Produkt von Stimulationsstrom und -zeit (Pulsphasenzeit), angegeben in der Maßeinheit nC mit

$$\boldsymbol{Q}=\boldsymbol{I}\cdot \boldsymbol{t}$$

Wenn diese Maßeinheit nicht verfügbar ist, soll die herstellerabhängige Intensitätseinheit angegeben und entsprechend gekennzeichnet werden.
